# Treating diagnostic frameworks as information systems: normative implications and opportunities

**DOI:** 10.1038/s41398-026-04369-9

**Published:** 2026-11-04

**Authors:** Philip Millroth

**Affiliations:** Department of Psychology, Uppsala University, Uppsala, Sweden

**Keywords:** Scientific community, Health sciences

## Diagnostic frameworks as information systems: normative implications and opportunities

The DSM-5 and ICD-11 are not just clinical manuals; they are information architectures. Every time a clinician applies diagnostic criteria, every time a researcher aggregates symptoms into a diagnostic variable, and every time a committee drafts a revision, they operate on the same basic object: a coded system for transmitting information about mental states. Yet no one has applied information theory [1] to evaluate the architecture of diagnostic systems themselves. Existing applications of information-theoretic thinking in psychiatry — model-selection criteria such as AIC and BIC — assess how well statistical models fit covariance data; they say nothing about the efficiency of the diagnostic code itself. This Viewpoint argues that information theory (henceforth, IT) — the elsewhere highly influential mathematical framework developed by Shannon to characterize the capacity and efficiency of communication systems [1] — provides what is currently missing: a principled, quantitative basis for designing, evaluating, and reforming diagnostic frameworks and the instruments that operationalize them.

The case for this reframing is not merely theoretical. Diagnostic quality, the product of validity and reliability in the DSM-5 and ICD-11 is, by available estimates, remarkably poor -essentially entailing that the majority of variability stems from measurement error or other irrelevant factors [2, 3].

## Diagnoses as codes, symptoms as signal

In the IT framework, an information source generates signals that pass through a channel to a receiver; the channel’s capacity depends on the statistical structure of the signals and the noise in the transmission [1]. Psychiatric diagnosis maps cleanly onto this picture. The symptom space is the source, diagnostic categories are the encoding, and clinical or research decisions are the receiver. Each key IT quantity translates directly into a diagnostic design question (Table 1). The entropy of a diagnosis — the uncertainty in its symptom profile — quantifies how heterogeneous, and therefore how informationally diffuse, the construct is. Mutual information between two diagnoses quantifies their genuine overlap beyond surface similarity. Channel capacity sets the theoretical maximum for how precisely the system can ever discriminate among conditions, regardless of clinician expertise.

**Table 1:** Core Information-Theoretic Concepts and their Interpretation in Diagnostic System Design.

IT concept	Diagnostic interpretation	Design implication
Entropy of a diagnosis	Uncertainty in the symptom profile of a diagnosis; high values indicate heterogeneous, informationally diffuse constructs	High-entropy diagnoses carry less diagnostic signal per criterion
Mutual information between diagnoses	Shared symptom information beyond surface wording; captures genuine structural overlap	Excess mutual information inflates observed comorbidity
Conditional entropy of diagnosis given a symptom	Diagnostic uncertainty remaining after a symptom is observed	Low values identify symptoms with high discriminative value
Redundancy	Fraction of diagnostic bandwidth occupied by non-unique information	Drives within-category heterogeneity and between-category overlap
Channel capacity	Theoretical maximum discriminability of the diagnostic system	Sets the ceiling on diagnostic precision regardless of clinician expertise

## The redundancy problem, quantified

A recent descriptive study makes the value of this framework concrete. Forbes and colleagues [4] mapped the 1419 symptom listings across 202 DSM-5 diagnoses and identified 628 distinct symptoms. Of these, 231 (36.8%) repeat across multiple diagnoses, and these repeating symptoms account for many symptom listings — meaning that a vast amount of what clinicians encounter in the diagnostic criteria is, informationally, non-unique. Symptoms of major depressive disorder are among the most pervasive in the DSM‑5: 10 of the 15 most non‑specific symptoms are MDD criteria, and insomnia alone appears in 22 diagnoses across 8 chapters [4]. From an IT perspective, this is a structural encoding failure — a channel that reuses the same codewords for different messages, systematically reducing its capacity to discriminate among them. The well-documented consequences — inflated comorbidity driven by shared criteria, within-category heterogeneity that obscures causal mechanisms, and poor predictive validity for treatment and biomarkers [5] — are precisely the failures IT predicts from an inefficient code.

## A normative framework, not just a descriptive one

The value of IT here is not that it measures redundancy, but that it specifies what an efficient diagnostic code would look like. The framework yields concrete, actionable design principles [1]. A symptom with high conditional entropy given the diagnosis it belongs to is doing informational work; a symptom with low conditional entropy is contributing noise or replicating information already encoded by other criteria. Mutual information between diagnoses should reflect genuine co-occurrence of underlying processes, not shared wording. A well-designed diagnostic system should represent maximal psychopathological variation with minimal redundancy. Consider insomnia as an illustration. Listed in 22 diagnoses across 8 DSM-5 chapters, its conditional entropy given any single diagnosis is near-zero: observing insomnia conveys almost no information about which diagnosis is present. An IT audit would flag it not for removal but for reclassification — either as an explicit transdiagnostic marker carrying its own specification, or as a non-contributing criterion whose weight in polythetic sets should be reduced. The design decision becomes empirically grounded rather than committee-driven.

These principles extend naturally to the measurement instruments through which diagnostic systems are operationalized. Structured clinical interviews are themselves information architectures: their item selection, branching logic, and decision thresholds collectively determine the channel capacity of the assessment — how much diagnostic signal survives the measurement process. An IT framework provides a principled basis for evaluating whether a given instrument’s items are recovering independent signal or recapturing information already encoded in prior items, and for optimizing interview structure accordingly.

Some limitations deserve acknowledgment. IT identifies where a diagnostic code is inefficient but does not prescribe solutions — deciding what to do about a low-entropy symptom requires clinical and theoretical judgment beyond the framework. The framework also treats symptoms as discrete, stable signals, which does not capture graded severity or context-dependence. These constraints bound its scope but do not undermine the core claim: that coding efficiency is a legitimate and currently unmeasured dimension on which diagnostic systems should be evaluated. Finally, the framework is sometimes read as presupposing a unified causal ontology — as if low entropy implied that a symptom should map onto a single underlying process. It does not. Shannon’s framework evaluates the properties of a code, not the etiology of what is being coded. A symptom may appear across 22 diagnoses precisely because it is a downstream consequence of multiple distinct causal pathways; IT captures that non-specificity without taking any position on what produces it.

## Implications for reform

Current proposals for reforming psychiatric classification — such as the Hierarchical Taxonomy of Psychopathology (HiTOP) [6] and the NIMH Research Domain Criteria (RDoC), as well as recent integrative syntheses of the two [7] — share a dissatisfaction with the DSM’s categorical structure but lack a unifying formal criterion for what a better system should look like. HiTOP derives structure from patterns of comorbidity and symptom covariation; RDoC anchors to neurobiological constructs. The reform of ICD-11 has made similar incremental adjustments [3]. All are empirically motivated but atheoretical with respect to the information structure of the resulting classification. Recent work applying combinatorial set theory to identify minimally sufficient symptom sets [8], converges on compatible conclusions from a different formal angle, reinforcing that the structural problems in diagnostic criteria are tractable through rigorous quantitative analysis. An IT framework supplies the missing criterion. Entropy and mutual information can be computed directly on any diagnostic system given a symptom-by-diagnosis incidence matrix, enabling direct empirical comparison of DSM-5, ICD-11, HiTOP, and proposed alternatives on a common metric.

This is the crucial point. Diagnostic systems have long been evaluated by their fit to covariance data, including through information-theoretic model-selection criteria, their clinical utility, and their acceptability to stakeholders. What has not been attempted is evaluating the information-architectural efficiency of the code itself: how much diagnostic signal survives the encoding, how much bandwidth is consumed by redundancy, and what ceiling the system places on discriminability regardless of how well any latent model fits. Information theory opens that line of evaluation. Treating psychiatric diagnosis as an information architecture is not a metaphor, but a formalization that allows the intuitions behind decades of critique to be made precise, their consequences derived, and the next generation of diagnostic tools designed with efficiency and discriminability as explicit objectives.
